# Intraneuronal Aβ detection in 5xFAD mice by a new Aβ-specific antibody

**DOI:** 10.1186/1750-1326-7-8

**Published:** 2012-03-16

**Authors:** Katherine L Youmans, Leon M Tai, Takahisa Kanekiyo, W Blaine Stine, Sara-Claude Michon, Evelyn Nwabuisi-Heath, Arlene M Manelli, Yifan Fu, Sean Riordan, William A Eimer, Lester Binder, Guojun Bu, Chunjiang Yu, Dean M Hartley, Mary Jo LaDu

**Affiliations:** 1 Department of Anatomy and Cell Biology, University of Illinois at Chicago, Chicago IL 60612, USA; 2 Department of Neuroscience, Mayo Clinic, Jacksonville FL 32224, USA; 3 Department of Medicine, Division of Geriatrics, Evanston Northwestern Healthcare Research Institute, Evanston Illinois 60201, USA; 4 Department of Neurological Sciences, Rush University Medical Center, Chicago IL 60612, USA; 5 Department of Cell and Molecular Biology, Feinberg School of Medicine, Northwestern University, Chicago IL 60611, USA; 6 CMC Program Management, Global Pharmaceutical Operations, Abbott Bioresearch Center, 100 Research Drive, Worcester MA 01605, USA; 7 Neuroscience Research, Global Pharmaceutical Research and Development, Abbott, 100 Abbott Park Road, Abbott Park IL 60064-6123, USA

**Keywords:** Intraneuronal, Aβ, APP, MOAB-2, 3xTg, 5xFAD, Antibody, Alzheimer's disease

## Abstract

**Background:**

The form(s) of amyloid-β peptide (Aβ) associated with the pathology characteristic of Alzheimer's disease (AD) remains unclear. In particular, the neurotoxicity of intraneuronal Aβ accumulation is an issue of considerable controversy; even the existence of Aβ deposits within neurons has recently been challenged by Winton and co-workers. These authors purport that it is actually intraneuronal APP that is being detected by antibodies thought to be specific for Aβ. To further address this issue, an anti-Aβ antibody was developed (MOAB-2) that specifically detects Aβ, but not APP. This antibody allows for the further evaluation of the early accumulation of intraneuronal Aβ in transgenic mice with increased levels of human Aβ in 5xFAD and 3xTg mice.

**Results:**

MOAB-2 (mouse IgG_2b_) is a pan-specific, high-titer antibody to Aβ residues 1-4 as demonstrated by biochemical and immunohistochemical analyses (IHC), particularly compared to 6E10 (a commonly used commercial antibody to Aβ residues 3-8). MOAB-2 did not detect APP or APP-CTFs in cell culture media/lysates (HEK-APP_Swe _or HEK-APP_Swe_/BACE1) or in brain homogenates from transgenic mice expressing 5 familial AD (FAD) mutation (5xFAD mice). Using IHC on 5xFAD brain tissue, MOAB-2 immunoreactivity co-localized with C-terminal antibodies specific for Aβ40 and Aβ42. MOAB-2 did not co-localize with either N- or C-terminal antibodies to APP. In addition, no MOAB-2-immunreactivity was observed in the brains of 5xFAD/BACE^-/- ^mice, although significant amounts of APP were detected by N- and C-terminal antibodies to APP, as well as by 6E10. In both 5xFAD and 3xTg mouse brain tissue, MOAB-2 co-localized with cathepsin-D, a marker for acidic organelles, further evidence for intraneuronal Aβ, distinct from Aβ associated with the cell membrane. MOAB-2 demonstrated strong intraneuronal and extra-cellular immunoreactivity in 5xFAD and 3xTg mouse brain tissues.

**Conclusions:**

Both intraneuronal Aβ accumulation and extracellular Aβ deposition was demonstrated in 5xFAD mice and 3xTg mice with MOAB-2, an antibody that will help differentiate intracellular Aβ from APP. However, further investigation is required to determine whether a molecular mechanism links the presence of intraneuronal Aβ with neurotoxicity. As well, understanding the relevance of these observations to human AD patients is critical.

## Background

The form(s) of amyloid-β peptide (Aβ), particularly the 42 amino acid form (Aβ42), associated with the neurotoxicity characteristic of Alzheimer's disease (AD) remains unclear. The potential toxic assemblies of the peptide include soluble Aβ [1], oligomeric Aβ [2], intraneuronal Aβ [3] and specific plaque morphology [4]. Evidence indicates that intraneuronal Aβ accumulation may be an important proximal neurotoxic event in AD pathogenesis (reviewed in [5,6]). Studies suggest intraneuronal Aβ accumulation in AD [7-9] and Down's Syndrome patients [10,11]. However, the relationship between intraneuronal Aβ and plaque deposition remains unclear. Evidence suggests that intraneuronal Aβ may precede extracellular plaque deposition in the brains of AD patients [12,13]. In particular, intraneuronal Aβ42 accumulates in AD susceptible brain regions and precedes both extracellular amyloid deposition and neurofibrillar tangle formation [3]. The "inside-out" hypothesis posits that the intraneuronal Aβ remaining after neuronal apoptosis serves as seeds for amyloid plaques. This is supported by several human studies demonstrating that increasing plaque deposition corresponds to decreased intraneuronal Aβ staining [8,9]. However, beyond this temporal sequence, the functional connection between the deposition of Aβ in neurons and the parenchyma has not been established in human brain.

To further investigate intraneuronal Aβ, attention has focused on analysis of transgenic mice with increased levels of human Aβ (Aβ-Tg mice). In accordance with data from AD patients, intraneuronal Aβ precedes plaque deposition in multiple Aβ-Tg mouse models ([14-23]) and may decrease as plaque deposition increases ([17,19,22,24]). Importantly, clearance of intraneuronal Aβ via immunotherapy reversed cognitive deficits in triple-transgenic (3xTg mice) mice that harbor the PS1_M146V_, APP_Swe _and tau_P301L _transgenes [14,19]. Furthermore, after termination of immunotherapy, intraneuronal Aβ re-appears prior to extracellular plaque deposition [20]. Intraneuronal Aβ is also associated with impaired long-term potentiation (LTP), cognitive deficits and eventual neuronal loss in Aβ-Tg mouse models ([14,15,17-19]).

However, the neurotoxicity of intraneuronal Aβ accumulation is an issue of considerable controversy; indeed even the existence of Aβ deposits within neurons is currently subject to debate and interpretation http://www.alzforum.org/res/for/journal/detail.asp?liveID=193. Concern centers on whether the detected intraneuronal immunoreactivity is the result of Aβ antibodies binding to APP [16]. Recently, Winton and co-workers used 3xTg mice to demonstrate intraneuronal immunodetection with the commonly used commercial antibodies 6E10 (residues 3-8 of Aβ), 4G8 (residues 17-24 of Aβ) and 22C11 (N-terminal APP residues 66-81), but not with C-terminal Aβ40- and 42-specific antibodies [25]. This staining pattern was unchanged in the absence of Aβ (3xTg/β-secretase (BACE)^-/- ^mice), suggesting the intraneuronal staining represents APP and not Aβ. These data are in stark contrast to multiple publications demonstrating intraneuronal Aβ staining in 3xTg mice and other Aβ-Tg mice [14,19,20,26].

These issues highlight experimental considerations that need to be addressed in order to investigate intraneuronal Aβ accumulation *in vivo*. First, as the conformation or conformations of intraneuronal Aβ is not known, the detection of intraneuronal Aβ it is likely to be optimal with a pan-specific antibody that detects different conformations of Aβ. Second, antibodies must be specific for Aβ and not detect APP. Thus, intraneuronal Aβ cannot be specifically identified by antibodies directed against residues 3-8 (e.g. 6E10), and residues 17-24 (e.g. 4G8) of Aβ because these antibodies also recognize full length APP [16] and APP C-terminal fragments (APP-CTFs) [27-30]. This is particularly relevant for Aβ-Tg mouse models that express high levels of the APP transgene (e.g. 2 and ~5 fold higher in the brains of the hemizygous and homozygous 3xFAD mice than endogenous APP in wild-type (WT) mice [19]). Third, the detection of intraneuronal Aβ in Aβ-Tg mouse models can be confirmed by genetic or pharmacological approaches. For example, in Tg-ArcSwe/BACE1^-/- ^mice and Tg-ArcSwe mice treated with a γ-secretase inhibitor, no intraneuronal Aβ was detected with antibody 82E1 (Aβ residues 1-5) and Aβ42- and Aβ40-specific antibodies [31]. Fourth, co-localization with an intraneuronal organelle marker would provide further evidence for Aβ pathology exists within a neuron, distinct from Aβ associated with the cell membrane or in the extracellular space.

Due to this cross reactivity of anti-Aβ antibodies with APP, a mouse monoclonal antibody (MOAB-2, IgG2_b_) was developed that is specific for Aβ and does not detect APP. MOAB-2 is a pan-specific monoclonal antibody that recognizes unaggregated (U), oligomeric (O), and fibrillar (F) forms of synthetic Aβ42, as well as unaggregated Aβ40. MOAB-2 did not detect APP or APP-CTFs in cell culture media/lysates (HEK-APP_Swe _or HEK-APP_Swe_/BACE1) or in brain homogenates from transgenic mice expressing 5 FAD mutations (5xFAD mice) [18]. By immunohistochemistry (IHC) analysis of 5xFAD brain tissue, MOAB-2 co-localized with Aβ40- and 42 C-terminal specific antibodies, but does not co-localize with N- or C-terminal APP antibodies. No MOAB-2 immunoreactivity was observed in the brains of 5xFAD/BACE^-/- ^mice although significant amounts of APP were detected with anti-APP antibodies as well as by 6E10. In both 5xFAD and 3xTg mouse tissue, MOAB-2 co-localized with cathepsin-D, a marker for acidic organelles, providing further evidence for specifically identifying intraneuronal Aβ. In addition, MOAB-2 demonstrated strong intraneuronal and extra-cellular immunoreactivity in 5xFAD and 3xTg brain tissue.

## Results

### Biochemical characterization: Identifying the MOAB-2 epitope on Aβ

Peptide arrays consisting of a series of overlapping 10-mers from the -4 position of the Aβ sequence to residue 46 were used to identify the epitope of MOAB-2 (Additional file 1: Figure S1A). Using these membranes, MOAB-2 detection was exclusive to residues 1-6, as this is the only sequence common to the 5 overlapping 10-mers detected by MOAB-2. As shown, the IgG_2b _negative control was blank.

Taking advantage of the sequence difference between human and rat/mouse Aβ, which includes a difference at residue 5 (arginine in human, glycine in rodent), the MOAB-2 epitope was further refined to residues 1-4 of Aβ (Additional file 1: Figure S1B). By Dot blot, MOAB-2 detected rat Aβ40 and human Aβ40, albeit with less affinity than for Aβ42 (discussed below). The sequence for rat Aβ and human Aβ40 are given below, with the differences at positions 5, 10 and 13 in bold.

Rat Aβ**: **DAEF**G**HDSG**F**EV**R**HQKLVFFAEDVGSNKGAIIGLMVGGVV

Human Aβ: DAEF**R**HDSG**Y**EV**H**HQKLVFFAEDVGSNKGAIIGLMVGGVV

### Biochemical characterization: MOAB-2 detects Aβ40 and multiple conformations of Aβ42 at low antigen and antibody concentrations

**Figure 1:**
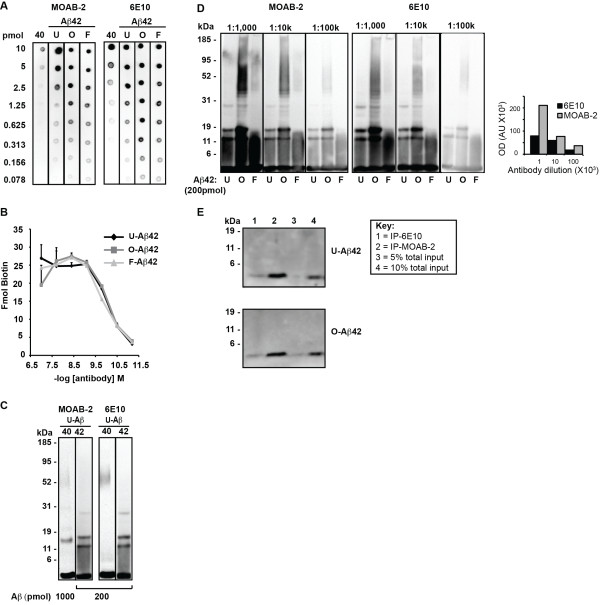
**MOAB-2 detects multiple Aβ conformations at low antibody concentrations**. **(A) **Dot blot of serial Aβ40 (unaggregated) and Aβ42 dilutions (unaggregated (U), oligomeric (O) or fibrillar (F)) probed with MOAB-2 or 6E10. **(B) **Antibody-binding saturation curve of 25 ng Aβ42 in the different aggregation states (U, O, and F) immobilized on microtiter plates, incubated with different MOAB-2 concentrations as indicated on figure. **(C) **Western-blot analysis of 1000 pmol U-Aβ40 or 200 pmol U-Aβ42 probed with MOAB-2 (lanes 1-2) or 200 pmol U-Aβ40 and U-Aβ42 probed with 6E10(lanes 3-4). (**D) **Western-blot analysis comparing antibody dilutions of MOAB-2 and 6E10 (0.5 mg/ml antibody stock concentrations) using U-, O- and F-Aβ42 (200 pmol). Quantification of Aβ immunoreactivity for each antibody dilution estimated using image J. **(E) **Immunoprecipitation of U- and O-Aβ42 (0.5 pmol) with 6E10 or MOAB-2 (Lane 1 = IP-6E10, 2 = IP-MOAB-2, 3 = 5% total input (0.025 pmol), 4 = 10% total input (0.05 pmol). The dilution of MOAB-2 for Figures 1A and C is 1:5000 from a stock of 0.5 mg/ml.

Recent research indicates that the role of Aβ42 in neurotoxicity may be dependent on the conformation of the peptide aggregates. Thus, to investigate Aβ accumulation *in vivo*, it is useful for an anti-Aβ antibody to detect multiple assembly states Aβ but not APP. Previously, an assembly protocol was optimized to produce preparations enriched in unaggregated (U), oligomeric (O), and fibrillar (F) forms of synthetic Aβ42 [32]. Under the conditions of this protocol, Aβ40 remained unaggregated. As assessed by dot blot (Figure 1A), MOAB-2 detects preparations enriched in U-, O-, F-Aβ42, and U-Aβ40 [32,33], and is thus a pan-specific Aβ antibody. However, MOAB-2 is selective for the more neurotoxic Aβ42 compared to Aβ40. Indeed, MOAB-2 demonstrated a titration against antigen concentration, and detects Aβ40 at 2.5 pmol but U-, O- and F-Aβ42 at antigen concentrations as low as ~ 0.1 pmol (Figure 1A). The commercial Aβ antibody 6E10 (antibody to residues 3-8 of Aβ appeared less selective for Aβ42 vs. Aβ40 but exhibited antigen detection of Aβ42 comparable to MOAB-2 (Figure 1A). In addition to antigen concentration, MOAB-2 demonstrated an antibody-dependent saturation curve (Figure 1B) to a fixed amount of immobilized U-, O- or F-Aβ42 (25 ng). The EC_50 _values for MOAB-2 were not significantly different for U-, O- or F-Aβ42 conformations (13, 12 and 18 ng/ml respectively).

The ability of MOAB-2 to detect different molecular weight Aβ assemblies was assessed via Western blot analysis of proteins separated by SDS-PAGE. Given the apparent selectivity of MOAB-2 for Aβ42 versus Aβ40, 5-fold greater Aβ40 (1000 pmol) than Aβ42 (200 pmol) was loaded for comparable detection with MOAB-2. MOAB-2 and 6E10 identified bands corresponding to the size of Aβ42 monomer, trimer and tetramer with U-Aβ42 (Figure 1C). Aβ40 was predominantly monomeric, with a minor band consistent with tetramer detected with MOAB-2. In contrast, 6E10 detection of Aβ40 (200 pmol) and U-Aβ42 (200 pmol) was comparable. To further assess sensitivity of MOAB-2 and 6E10, U-, O- and F-Aβ42 conformations were analyzed again by Western analysis using a wide range of antibody concentrations (500-5 ng/ml) (Figure 1D, left). MOAB-2 titrates with antibody concentration and multiple Aβ42 conformations were detected at MOAB-2 concentrations of 5 ng/ml. While 6E10 detected Aβ42 over the same antibody dilution range, the signal intensity was lower than MOAB-2. Comparing the optical densities of the 3 antibody dilutions using MOAB-2 and 6E10 highlights this difference (Figure 1D, right). In addition, immunoprecipitation with MOAB-2 resulted in a high recovery of Aβ42 (Figure 1E), equivalent to more than 10% of total input for both U- and O-Aβ42, and significantly higher than that of 6E10.

### Biochemical characterization: MOAB-2 does not detect APP/APP-CTFs in cell culture media and lysates or cortical brain extracts from 5xFAD mice

**Figure 2:**
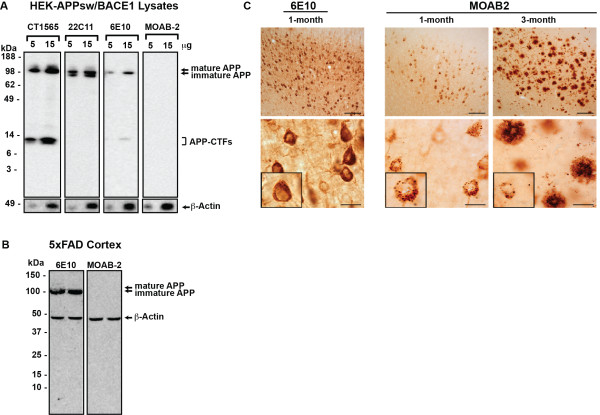
**MOAB-2 does not detect APP in cell culture media and lysates or cortical brain extracts from 5xFAD mice**. **(A) **Western-blot analysis of 5 μg or 15 μg cell lysates from HEK-APP_Swe_/BACE1 cells, probed with antibodies against C-terminus of APP (CT1565), N-terminus of APP (2211), Aβ (6E10, MOAB-2) or β-Actin (loading control). **(B) **Western analysis of 25 μg total protein from detergent-extracted 5xFAD mouse cortex probed with 6E10 or MOAB-2 and β-Actin for loading control. **(C) **DAB staining with 6E10 or MOAB-2 in 1- and 3-month old 5xFAD mice. Box insert: single neuron staining (Coronal sections, top = 10x scale, bar 100 μm; bottom = 100x, scale bar 20 μm). The dilution of MOAB-2 for Figures **2**A,B and C is 1:5000 from a stock of 0.5 mg/ml. (See "Dot- and Western-blot Analyses" in Methods for other antibody concentrations.).

A major issue for detecting Aβ *in vitro *and *in vivo *is that some Aβ antibodies recognize APP or C-terminal fragments of APP (APP-CTFs) [34-36]. Therefore to assess cross-reactivity of MOAB-2 with APP and APP-CTFs, HEK cells co-transfected to express APP_Swe _and BACE1 (HEK-APP_Swe_/BACE1) were used as these cells produce a substantial amount of APP-CTFs (Figure 2A) [37]. Western analysis demonstrates that an APP C-terminal antibody (CTF1565), 22C11 and 6E10 detect a ~100 kDa band consistent with APP, while MOAB-2 does not (Figure 2A). Importantly, CTF1565 and 6E10 also recognize ≤ 15 kDa bands consistent with APP-CTFs, while MOAB-2 does not.

To confirm that MOAB-2 does not recognize APP in brain homogenates, 5xFAD mouse cortex was extracted with 1% Triton X-100 [38], run on SDS-PAGE and analyzed by Western blot with 6E10 and MOAB-2 (Figure 2B). 6E10 detected a protein with a molecular weight consistent with APP that was not recognized by MOAB-2.

### Immunohistochemical (IHC) analysis: Staining in 5xFAD brain sections

Initially, to determine whether MOAB-2 would be effective at detecting Aβ by IHC, coronal sections of the frontal cortex from 1- and 3-month old 5xFAD mice [18] were immunostained with 6E10 and MOAB-2 and visualized via DAB staining (Figure 2C). In the frontal cortices of these mice at 1-month of age, 6E10 is strongly immunoreactive across the field of the cortex, while higher magnification shows that the cytoplasm is evenly stained with an immunonegative nuclei. In contrast, MOAB-2 staining of the cortical field is substantially less than for 6E10 and the intraneuronal staining is punctate (Figure 2C). These results are consistent with 6E10 detection of APP and Aβ, and MOAB-2 recognition of only Aβ. In 3-month old mice, extensive MOAB-2 immunopositive extracellular staining is consistent with increased plaque deposition. Higher magnification reveals low levels of MOAB-2 intraneuronal immunoreactivity with significant staining of individual plaques. As intraneuronal MOAB-2 immunoreactivity was detected at 1-month of age in 5xFAD mice, this age was used for subsequent experiments to determine the specificity of MOAB-2.

### IHC analysis: MOAB-2 detection of intraneuronal Aβ but not intraneuronal APP in 5xFAD brain tissue

**Figure 3:**
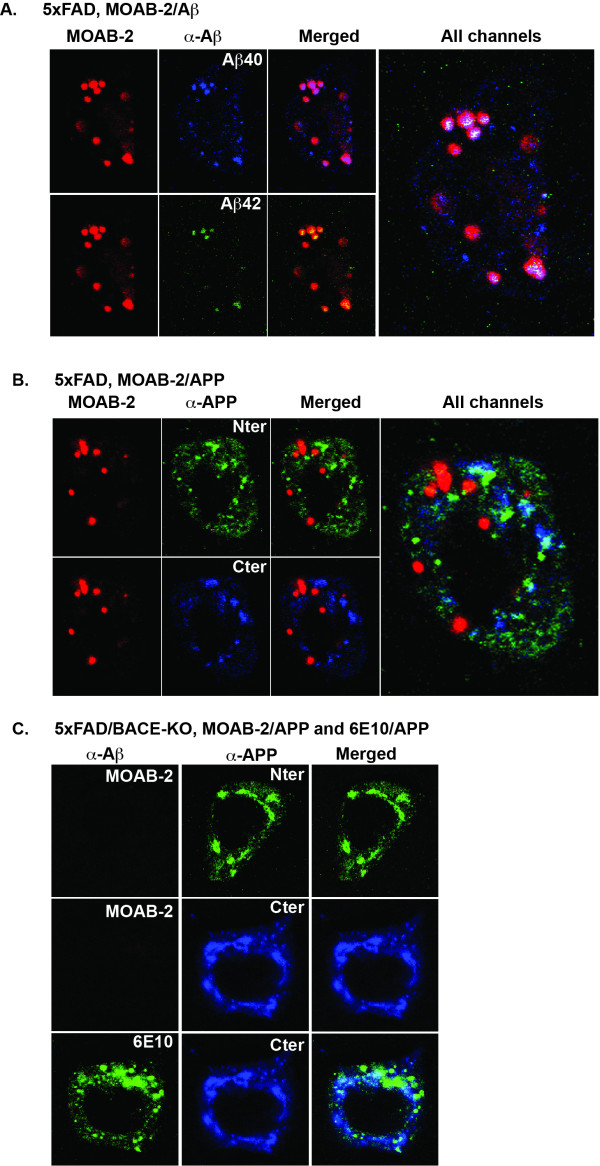
**MOAB-2 detection of intraneuronal Aβ but not intraneuronal APP in 5xFAD brain tissue**. Immunofluorescent detection of Aβ and APP in the cortex of: **(A) **1-month old 5xFAD mice using: MOAB-2, Aβ42- or Aβ40-specific antibodies; **(B) **1-month old 5xFAD mice with MOAB-2 and antibodies against the C-terminus (Cter) (CT695) or N-terminus (Nter) (2211) of APP; and **(C) **4-month old 5xFAD/BACE^-/- ^with 6E10 or MOAB-2, plus antibodies CT695 and 22C11. (Coronal sections, representative confocal images at 100x). Antibody concentrations: MOAB-2 (1:1000), anti-Aβ40, (1:1700), anti-Aβ42 (1:200), 22C11 (1:40), CT695 (1:500), 6E10 (1:1000).

For IHC detection of intraneuronal Aβ, the specificity of MOAB-2 for Aβ and APP was determined using immunofluorescent confocal microscopy. Coronal sections of the frontal cortex from 1-month old 5xFAD mice were co-immunostained with MOAB-2 and Aβ42- or Aβ40-specific antibodies (Figure 3A). Both the Aβ42- and Aβ40-specific antibodies demonstrate punctate intraneuronal immunoreactivity that co-localized with MOAB-2. Thus, MOAB-2 appears to detect intraneuronal Aβ.

To determine whether MOAB-2 staining cross-reacted with APP, coronal sections of the frontal cortex from 1-month old 5xFAD mice were co-stained MOAB-2 or 22C11 (N-terminus of APP) or CT695 (C-terminus of APP). MOAB-2 staining was punctate and did not co-localize with either APP antibodies (Figure 3B). The specificity of MOAB-2 for Aβ was confirmed via a genetic approach, utilizing brain tissue from 5xFAD/BACE^-/- ^mice that produce APP but not Aβ (Figure 3C). Significant immunoreactivity was observed with 22C11 and CT695, while no immunoreactivity was observed with MOAB-2 in the cortex of 4-month old animals. In contrast, 6E10 immunoreactivity co-localized with CT695, confirming 6E10 detection of APP.

### IHC analysis: MOAB-2 co-localization with cathepsin-D in 5xFAD and 3xTg brain tissue

**Figure 4:**
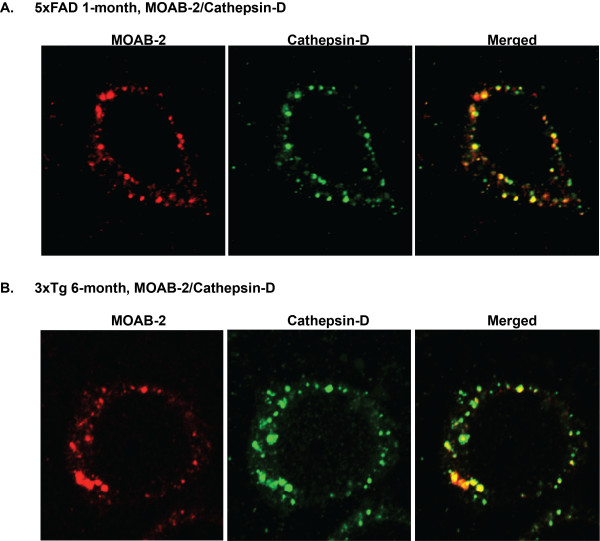
**MOAB-2 co-localization with cathepsin-D in 5xFAD and 3xTg mouse brain tissue**. Immunofluorescent detection of intraneuronal Aβ with MOAB-2 and an antibody against cathepsin-D (1:50 dilution) in the cortex of: **(A) **1-month old 5xFAD mice; and **(B) **6-month old 3xTg mice. (Coronal sections, representative confocal images at 100x).

Overall, the *in vitro *or *in vivo *data presented in Figures 1, 2, 3 demonstrate that MOAB-2 detects Aβ but not APP. In particular, intraneuronal MOAB-2 immunoreactivity is consistent with Aβ and does not appear to be due to cross-reactivity with APP. In cortical tissue from 1-month old 5xFAD (Figure 4A) and 6-month old 3xTg (Figure 4B) mice, MOAB-2 co-localized with cathepsin-D, a marker for lysosomes and other acidic organelles. Co-localization of MOAB-2 with an intracellular organelle marker provides further evidence of Aβ localization within a neuron, distinct from Aβ associated with the cell membrane or in the extracellular space. The majority of cells in the cortex were cathepsin-D immunopositive, as expected, whereas fewer cells were immunopositive for MOAB-2. In the cells that contained intraneuronal Aβ, while the majority of the cathepsin-D co-localized with MOAB-2, some cathepsin-D staining did not co-localize, consistent with not all lysosomes containing Aβ.

### IHC analysis: MOAB-2 detection of intraneuronal Aβ and extracellular plaques in 5xFAD and 3xTg mouse brain tissue

**Figure 5:**
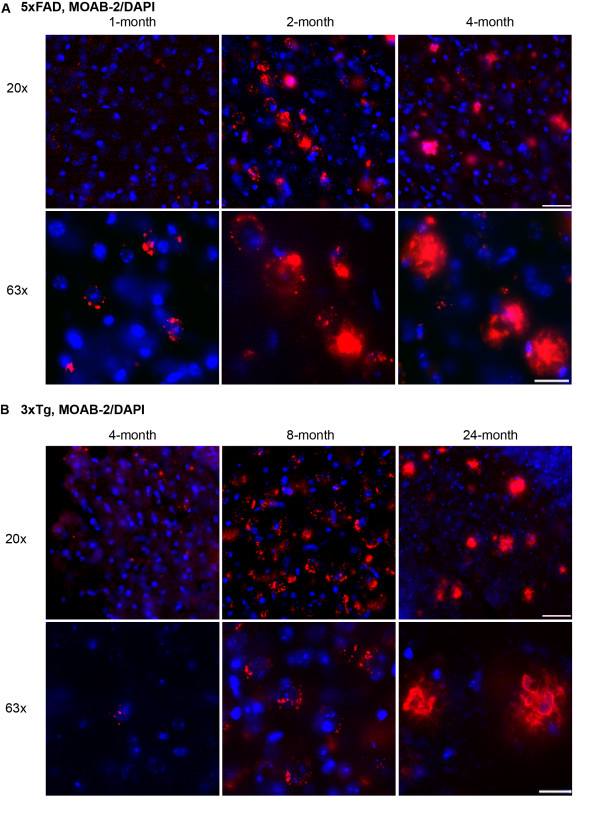
**MOAB-2 detection of intraneuronal Aβ and extracellular plaques in 5xFAD and 3xTg mouse brain tissues**. Immunofluorescent detection of Aβ with MOAB-2 in the subiculum of **(A) **1-, 2- and 4-month old 5xFAD mice and **(B) **4-, 8- and 24-month old 3xTg mice. (Coronal sections, representative fluorescent images, top = 20x, scale bar 50 μm; bottom = 63x, scale bar 20 μm).

Previous studies have demonstrated that intraneuronal Aβ accumulates prior to extracellular plaque deposition and decreases as plaque deposition increases. However, if the Aβ antibodies also detect APP, interpretation of the results can be problematic, as recently questioned by Winton and co-workers [25]. Compared to other Aβ-Tg mice such as 5xFAD mice (reviewed [39]), this concern is particularly relevant to the 3xTg mice as prominent intraneuronal Aβ staining is observed for an extended period of time, approximately 4- to 18-months [19]. As MOAB-2 detects intracellular Aβ and not APP, the progression of Aβ pathology was determined by IHC in the subiculum of 5xFAD and 3xTg mice (Figures 5A and 5B, respectively). 5xFAD mice exhibit accelerated Aβ pathology, with intraneuronal Aβ increased from 1- to 2-months and decreased by 4-months, while plaque deposition increased from 2- to 4-months. To 'match' the progression of Aβ pathology with 5xFAD mice, tissue sections from 4-, 8- and 24-month old 3xTg mice were stained with MOAB-2. Intraneuronal Aβ increased from 4- to 8-months and decreased by 24-months, while extracellular Aβ increased from 8- to 24-months. Intraneuronal Aβ deposition in the 3xTg mice is present over a broad age range prior to the deposition of extracellular Aβ. Thus 3xTg mice represent a model of Aβ pathology with intraneuronal the major site for accumulation of Aβ.

## Discussion

The identification of Aβ as the major component of amyloid plaques has led to the amyloid cascade hypothesis and the idea that reducing plaques would correlate with a reduction in AD symptoms [40]. However plaque load as detected post-mortem does not correlate with cognitive impairment pre-mortem (reviewed [41]). The amyloid cascade hypothesis has been modified [42], as attention shifted to soluble oligomeric Aβ conformations as the toxic form of the peptide. Soluble oligomeric Aβ has been detected from brain tissue [43] and demonstrated to correlate with cognitive deficits in AD patients (reviewed [41,44,45]). Oligomeric assemblies, including protofibril, annular assembly, 56* and dimer/trimers induce neurotoxicity *in vitro *and *in vivo *(reviewed [41,44,45]). While it is most likely that soluble Aβ assemblies and insoluble amyloid are in a form of dynamic equilibrium, it remains essential to consistently perform Aβ analyses *in vitro *and *in vivo*. MOAB-2 is a pan-specific monoclonal antibody (Aβ residues 1-4) that detects several conformational species of Aβ42 with high affinity via dot and Western blot, immunoprecipitates Aβ with a higher recovery compared to 6E10, and does not detect APP in cell culture lysates and brain homogenates from 5xFAD tissue. In addition to biochemical analysis, IHC staining with MOAB-2 demonstrates robust and specific intracellular Aβ immunoreactivity at low antibody concentrations in both 5x5AD and 3xTg mice.

In 3xTg mice, Winton and co-workers demonstrated intraneuronal APP detection by APP N- and C-terminal specific antibodies, as well as 6E10 and 4G8, in agreement with this study in 5xFAD mice. As 6E10 and 4G8 continue to be used to identify Aβ, both biochemically [46] and by IHC [27,47-49], these results underscore the importance of using antibodies that have been carefully characterized. MOAB-2 did not co-localize with either N- or C-terminal antibodies to APP, and MOAB-2-immunreactivity was not observed in the brains of 5xFAD/BACE^-/- ^mice, although significant amounts of APP were detected by N- and C-terminal antibodies to APP, as well as by 6E10. Winton and co-workers further conclude that intraneuronal Aβ cannot be detected using a panel of antibodies to the C-terminus of Aβ. However, confocal analysis with MOAB-2 demonstrated intraneuronal Aβ detection that co-localized with Aβ40- and Aβ42-specific antibodies, suggesting significant differences between the results of these two studies. While these differences could simply be due to the strain of transgenic mice (5xFAD versus 3xTg) or some unique Aβ epitope present in intraneuronal Aβ in 5xFAD mice, MOAB-2 co-localized with cathepsin-D, providing further evidence for the presence of intraneuronal Aβ. In addition, MOAB-2 demonstrated strong intraneuronal and extra-cellular immunoreactivity as pathology developed in the 5xFAD (1-4 months) and 3xTg (2-24 months) mouse brain tissue. This extracellular staining in the 3xTg mice is consistent with the study by Winton. Thus, the apparent differences between these two studies have no simple explanation. Contributing factors could include the method of microscopy, the panel of antibodies used, as well as genetic drift in both the transgenic mouse lines, 3xTg mice (F. LaFerla, personal communications) and 5xFAD mice, if not maintained by breeding 5xFAD hemizygous males with B6SJL F1 hybrid females (R. Vassar, personal communications; unpublished observations MJ LaDu and D. Hartley).

Intraneuronal Aβ accumulation is re-emerging as an important neurotoxic event in AD pathogenesis (reviewed in [39,50]). Reports from the early 1980's first described intraneuronal Aβ immunoreactivity in AD patients and non-demented control subjects [12,13]. However, this detection was assumed to represent cross reactivity with lipofuscin, tau or APP [16]. Subsequent studies in human tissue using Aβ42- or Aβ40-specific antibodies demonstrated intraneuronal Aβ immunoreactivity [7-9,51]. Further data demonstrates that Aβ aggregation can be initiated intracellularly, is primarily Aβ42 and accumulates in AD-susceptible brain regions, including the entorhinal cortex and hippocampus of AD patients compared to control subjects [7-9,51-53]. In humans, intraneuronal Aβ likely exists in a dynamic equilibrium with extracellular Aβ. The 'inside-out' hypothesis posits that some extracellular amyloid is seeded by the intraneuronal Aβ that remains following neuronal apoptosis [7-9,51].

Data from Aβ-Tg mouse models also support intraneuronal Aβ as is a potentially important component of AD pathology. Indeed, intraneuronal Aβ42 appears to cause neurodegeneration in transgenic mice expressing Aβ specifically targeted to the endoplasmic reticulum [54]. Consistent with the inside-out hypothesis, intraneuronal Aβ accumulation precedes plaque deposition in multiple Aβ-Tg mouse models including Tg2576 [21], APP_SL_/PS1 [23], 3xTg [14,19], 5xFAD [18], Tg-ArcSw [17] and APP/PS1KI [15], and as extracellular deposition increases, intraneuronal Aβ decreases [17,19,22,24]. A significant portion of data reporting accumulation and functionality of intraneuronal Aβ originates from the 3xTg mouse model. In 3xTg mice, intraneuronal accumulation is present at 3- to 4-months, persists until at least 12-months, and decreases from 12- to 18-months, as extracellular deposition increases from 6- to 24-months [14,19,26]. Therefore, 3xTg mice represent a model of substantial and sustained intraneuronal Aβ pathology. Indeed, after immunotherapy in 3xTg mice, intraneuronal Aβ reappears prior to extracellular plaque deposition [14,19,26] and levels of intraneuronal Aβ are associated with deficits in LTP [19] and cognitive impairment [14,19,26].

Despite the evidence demonstrating intraneuronal Aβ accumulation in both human AD patients and in Aβ-Tg mouse models, it remains unclear the extent to which intraneuronal Aβ contributes to neurodegeneration. In human tissue, detection of intraneuronal Aβ immunoreactivity is intermittent and not always associated with other measures of Aβ pathology [16]. In addition, the accumulation of intraneuronal Aβ during normal brain aging remains an unresolved issue because Aβ antibodies can cross react with APP and other APP metabolites [55]. If intraneuronal Aβ is not a significant event in human AD pathology, then the relevance of intraneuronal Aβ accumulation in Aβ-Tg mice is uncertain. Other elements specific to a particular Aβ-Tg mouse model may modulate neurotoxicity, making it difficult to assign causality to intraneuronal Aβ. For example, combinations of FAD mutations in APP and PS1 (and Tau_P301L_), and temporal links among multiple measures of pathology are two examples of interactions that prevent identification of factors specifically correlating with neurotoxicity [25,56,57]. Thus, the functional connection between intraneuronal Aβ deposits and neurodegeneration warrants further study, particularly in human subjects, both control and AD patients. Reagents such as MOAB-2 will facilitate future investigations.

## Conclusions

Although the importance of intraneuronal Aβ to AD pathology remains unclear, the ability to consistently detect these deposits with an Aβ-specific antibody is critical. MOAB-2 is specific for Aβ and demonstrates robust intraneuronal immunoreactivity *in vivo*. Thus, MOAB-2 has the potential to facilitate future investigations into the importance of intraneuronal Aβ, both in Aβ-Tg mouse models and human subjects.

## Methods

### Preparation of Aβ peptide

Aβ40 and Aβ42 peptides (California Peptide NAPA, CA) were prepared as previously described [32,33]. Briefly, the peptides were monomerized by dissolving to a final concentration of 1 mM in hexafluoroisopropanol (HFIP)(Sigma-Aldrich, St. Louis, MO), aliquoted into microcentrifuge tubes, the HFIP evaporated using a SpeedVac and the peptide was stored at -20°C until use. For assembly protocols, peptides were resuspended in dimethylsulfoxide (DMSO) to 5 mM and diluted to 100 μM in phenol red-free F12 media (BioSource, Camarillo, CA) for U- and O-Aβ42, or 10 mM HCL for F-Aβ42 assemblies, respectively. U-Aβ42 was freshly prepared just prior to use, O-Aβ42 preparations were aged for 24 hours at 4°C and F-Aβ42 preparations for 24 hours at 37°C. Previously, assembly protocols were optimized to produce preparations enriched in unaggregated, oligomeric or fibrillar forms of synthetic Aβ42. Under the conditions of this protocol, Aβ40 remained unaggregated [32].

Rat Aβ40 (Calbiochem, San Diego, CA) was resuspended in DMSO to 1 mM, and diluted to 100 μM in phenol red-free F12 media just prior to use.

### MOAB-2 generation

As previously described [58,59], female BALB/c mice were immunized with O-Aβ42 produced as outlined above. For the initial injection, the immunogen was suspended in 200 μl Complete Freund's Adjuvant at a concentration of 1 μg/μl. Subsequent subcutaneous injections of 200 μg immunogen in Incomplete Freund's Adjuvant were carried out until the serum titer of the mouse was half-maximal at a dilution of 2 × 10^-4 ^as judged by ELISA, with 50 ng of O-Aβ42 attached per well in the solid phase. Once the desired serum titer was attained, immune spleens were removed from the mice, dissociated, and fused with SP2/o myeloma cells. The resultant cell suspension was plated in 96 well plates, HAT selected and cultured for 10-14 days to allow clonal growth using standard hybridoma technology previously described [58-60]. Initial clonal selection was performed by antigen/antibody blotting. 5 mM O- or F-Aβ42 were incubated with Immobilon-P membrane at room temperature for 30 min. Following rinsing and blocking, hybridoma supernatant was spotted onto membrane with 96-pin replicator. Clonal supernatants from O-Aβ42-immunized mice that were positive on the O- membrane and F-Aβ42 membrane were selected for further subcloning. Mother clones were subcloned 3-4 times to assure monoclonality and to allow hybrids to stabilize. Antibodies were isotyped and the stable clones adapted to serum-free medium and placed in a bioreactor for antibody expression. Monoclonal antibodies were then purified to homogeneity using standard methods prior to storage at 1 mg/ml or 0.5 mg/ml in borate buffered saline containing 50% glycerol [58-60]. MOAB-2 (IgG_2b_) was a high titer antibody identified by this process.

### Source of antibodies

For the methods used in this study, the following primary antibodies were utilized: MOAB-2 (anti-Aβ, mouse IgG_2b_, 0.5 mg/ml), IgG_2b _(0.2 mg/ml, Sigma-Aldrich, St. Louis, MO), 6E10 anti-Aβ residues 3-8, mouse IgG_1_, 0.5 mg/ml; Covance, Princeton, NJ), 22C11 (anti-APP N-terminal, mouse IgG_1_, 1 mg/ml, Milipore, Billerica, MA), 4G8 anti-Aβ residues 17-24, mouse IgG, Senetek, Maryland Height, MD), CT1565 (anti-APP C-terminal, rabbit monoclonal #1565, 0.45 mg/ml, Epitomics, Burlingame, CA), CT695 (anti-APP C-terminal, rabbit polyclonal #51-2700, 0.25 mg/ml, Invitrogen, Carlsbad, CA), anti-Aβ40 (MM32-13.1.1, mouse IgG_1_, 1.8 mg/ml, Bu lab), anti-Aβ42 (rabbit, 0.35 mg/ml; Invitrogen, Carlsbad, CA), anti-β-actin (chicken, 1.0 mg/ml; Abcam, Cambridge MA) cathepsin-D (goat polyclonal, 0.2 mg/ml, Santa Cruz biotechnology, Santa Cruz, CA). The dilutions of each antibody stock are denoted in the appropriate Methods section or Figure Legend.

### Aβ peptide arrays

A peptide array (PepSpotsTM) consisting of a series of overlapping 10-mers from the -4 position of the Aβ sequence to residue 46 covalently bonded via the carboxyl terminus to a cellulose membrane was prepared by JPT Peptide Technologies, GmbH, Berlin, Germany and used according to the manufacturer's recommendations (generously provided by C. Glabe). Membranes (n = 3) were incubated with 100 ng/ml of MOAB-2 or IgG_2b _isotype matched control (Sigma-Aldrich, St. Louis, MO) and then rabbit-anti-mouse antibody conjugated with HRP and visualized with ECL substrate.

### Tissue preparation

For *in vitro *analysis of APP, cortex samples were extracted and homogenized as described [38]. 3xTg mouse tissue was obtained from F. LaFerla, University of California, Irvine CA. 5xFAD/BACE^-/- ^tissues were obtained from R. Vassar, Northwestern University, Chicago, IL.

The *in vivo *experiments described below follow either the Rush University Medical Center Institutional Animal Care and Use Committee protocols, or the UIC Institutional Animal Care and Use Committee protocols.

#### IHC -DAB protocol (these experiment were conducted at Rush University Medical Center)

Mice are housed under standard conditions with access to food and water *ad libitum*. 1-, 2-, and 4-month old 5xFAD mice and were anesthetized with a single injection of ketamine/xylazine (100 mg/kg/5.0 mg/kg) and transcardially perfused with 0.9% saline for 2 min followed 4% paraformaldehyde and 0.1% glutaraldehyde made in 0.1 M phosphate buffer (PB) for 4 min. Brains were removed and dissected at the midline. The right hemibrains were post-fixed in the same fixative for 24 hours at 4°C and then stored in 30% sucrose at 4°C. Hemibrains were frozen on dry ice and coronal sections were cut immediately at 40 μm thickness on a sliding microtome. Sections were stored in cryoprotectant (30% glycerin, 30% ethylene glycol, in 0.1 M PB) at -20°C until analysis.

#### IHC -fluorescence protocol (these experiments were conducted at UIC)

Mice were anesthetized with sodium pentobarbital (50 mg/kg) and transcardially perfused with ice-cold PBS + Protease Inhibitor Cocktail (Calbiochem, set 3). Directly following perfusion, brains were removed and dissected at the midline. Left hemibrains from mice at each age were drop-fixed in 4% paraformaldehyde (PFA) for 48 hrs followed by storage at 4°C in phosphate buffered saline (PBS) + 0.05% sodium azide (NaN_3_) until use. Prior to IHC, hemi-brains were dehydrated in 30% sucrose for 48 hours.

For tissue homogenization: Right hemibrains were rapidly dissected on ice into cortex (CX), hippocampus (H) and cerebellum (CB), immediately snap frozen in liquid nitrogen, and stored at -80°C until use.

### Cell culture

HEK-APP_Swe_/BACE1 cells (obtained from R.Vassar, Northwestern University, Chicago, IL) [37] were grown to confluence in DMEM medium supplement with 10% fetal bovine serum and 100 μg/ml of G418. Cells were washed twice with 1xPBS, and grown in DMEM medium for another 3 days. The conditioned medium was collected, and cell lysates were prepared in 1xRIPA buffer (Sigma-Aldrich) supplemented with 1x protease inhibitor mix (Calbiochem).

### Dot- and western-blot analyses

Gel electrophoresis and Western blot analyses were performed according to manufacturer's protocols (Invitrogen) as previously described [32,33]. Briefly, samples in lithium dodecyl sulphate (LDS) sample buffer (Invitrogen) were heated to 90°C for 5 minutes and loaded into wells of 12% or 4-12% Bis-TRIS NuPAGE gels, electrophoresed using MES running buffer and transferred to 0.45 μm PVDF membranes (Invitrogen). Membranes were blocked in 5% non-fat dry milk in Tris-buffered saline containing 0.0625% Tween-20 (TBST), and incubated in primary followed by HRP-conjugated secondary antibodies (1:5000 dilution of 0.8 mg/ml stocks, Jackson Immunoresearch, West Grove, PA). For *in vitro *analyses, the following antibody dilutions were used (unless otherwise noted on Figure or in Figure Legend): MOAB-2 (1:5000), 6E10 (1:5000), CT1565 (1:2500), 22C11 (1:500), and β-actin (1:5000). Immunoreactivity was detected using enhanced chemiluminescence (ECL; Amersham Pharmacia) and imaged on a Kodak Image Station 4000R. Molecular weight values were estimated using pre-stained molecular weight markers. For dot-blots samples were loaded onto 0.45 μm PVDF membranes through wells of a dot-blot apparatus, and washed with TBST buffer. All subsequent blocking and antibody incubation steps were performed as described for Western blot analysis. The amount of each Aβ peptide, cell lysates or tissue homogenate is specified on the appropriate Figure or in the Figure Legend.

### Immunoprecipitation

0.5 pmol of U- and O-Aβ42 were diluted in TBST buffer. Protein A/G agarose beads (UltraLink Immobilized Protein A/G, Pierce) was added to pre-clear non-specific association with the beads. 10 μl of 0.5 mg/ml MOAB-2 or 6E10 antibodies were incubated with Aβ42 at 4°C overnight. Protein A/G agarose beads were added for an additional 2 hr. After a brief centrifugation, the pellets of Aβ42/antibody/Protein A/G complex were washed thoroughly with TBST buffer at 4°C, and boiled for 5 min in 1xLDS buffer with 5% β-mercaptoethanol. Released Aβ42 was separated in 12.5% NuPAGE, 0.025 pmol and 0.05 pmol of Aβ42 were also included to gauge the immunoprecipitation efficiency. Aβ42 were analyzed by Western blotting with biotinylated 4G8 antibody/StreptAvidin-HRP pair and visualized by ECL (SuperSignal West Dura, Thermo Scientific), the gel image was captured by Kodak Image Station 4000R.

### Solid plate binding assay

MOAB-2 binding to Aβ was assessed by a solid plate binding assay as previously described [61]. 25 ng of U-, O-, and F-Aβ42 were immobilized onto microtiter plate wells in PBS for 2 hr. All the incubation steps were performed at 37°C. The wells were then blocked with 1% BSA in PBS for 1 hr, incubated for 1 hr with the primary antibody (MOAB2/control IgG), washed, and incubated for 1 hr with a biotinylated anti-IgG antibody. The binding was quantified by incubation with a streptavidin-Eu conjugate, measured by time resolved Eu-fluorescence and converted into fmols of biotin. Negative control (binding to BSA covered wells, with no Aβ) was subtracted from all the binding curves. EC50 values were calculated using non-linear curve fitting, GraphPad Prism version 4.00, GraphPad Software, San Diego California USA.

### Immunohistochemical analysis: Diaminobenzidine staining

Note: Initial characterization of MOAB-2 by IHC demonstrated no significant differences in Aβ detection using paraffin and free-floating sections (Bu and LaDu lab, data not shown). Formic acid (FA) treatment resulted in optimal detection of both intraneuronal and extracellular Aβ compared to without FA. This is consistent with data from Christensen and co-workers who demonstrated that FA was essential for IHC detection of aggregated intraneuronal Aβ in mouse models of AD, including 5xFAD [27]. Thus, FA was used for both DAB and immunofluorescent, as described below.

Tissues from 1- and 3-month old 5xFAD mice were processed as free-floating sections and immunostained using the mouse monoclonal antibodies 6E10 (1:1000) and MOAB-2 (1:550). All procedures were conducted at room temperature, except primary antibody incubation was done at 4°C. Briefly, 40 μm-thick coronal sections were rinsed in 0.1 M PBS (3 × 10 min), washed in TBS (3 × 10 min), incubated in 88% FA (8 min) for antigen retrieval [28], washed (TBS, 3 × 10 min) and incubated in 0.1 M sodium metaperiodate (20 min; Sigma-Aldrich) to quench endogenous peroxidase activity. Tissue sections were permeabilized in TBS containing 0.25% Triton X-100 (TBSX; 3 × 10 min), blocked with 3% horse serum in TBSX (3 × 10 min) followed by 1% horse serum in TBSX (2 × 10 min) and incubated with appropriate primary antibodies diluted in TBSX containing 1% horse serum overnight. Subsequently, sections were washed (1% horse serum, 3 × 10 min) incubated with biotinylated secondary antibody (anti-mouse IgG; 1:200, 1 hr Room temperature) washed (TBS 3 × 10 min) and then incubated with avidin-biotin complex (1:500; Elite Kit, Vector Labs) for 1 hr. Sections were washed in a 0.2 M sodium acetate trihydrate and 1.0 M imidazole solution (pH 7.4 with acetic acid; 3 × 10 min). Reaction products were visualized using an acetate-imidazole buffer containing 0.05% 3/3'-diaminobenzidine tetrahydrochloride (DAB; Sigma, MO) and 0.0015% hydrogen peroxide. For comparison purposes, sections immunostained with the same antibody were incubated in DAB for the same duration. Sections were then washed in acetate-imidazole buffer (3 × 5 min), transferred to TBS, mounted onto glass slides, air dried overnight, dehydrated through a series of graded alcohols (70%, 95%, 100%; 3 × 5 min), cleared in xylene (3 × 5 min) and cover-slipped with DPX (BDH Laboratory Supplies, Poole, UK).

### Immunohistochemical analysis: Immunofluorescent microscopy

Tissues were processed as outlined above, washed in TBS (3 × 10 min), incubated in 88% FA (8 min), permeabilized in TBSX (3 × 10 min), and blocked in TBSX containing 5% bovine serum albumin (BSA; 1 hr). Sections were subsequently incubated with appropriate primary antibodies diluted in TBSX containing 2% BSA overnight on an oscillatory rotator. For IHC analyses, the following primary antibodies were used: MOAB-2 (1:1000), anti-Aβ40, (1:1700), anti-Aβ42 (1:200), 22C11 (1:40), CT695 (1:500), 6E10 (1:1000) and cathepsin-D (1:50). The following day, sections were washed in TBSX (6 × 10 min), followed by Alexa fluorophore-conjugated secondary antibodies diluted 1:200. Images were captured on a Zeiss Axio Imager M1 under identical capture settings, at 20× or 63× magnification (as indicated) or at 100× with a Zeiss LSM 510 confocal microscope.

## Abbreviations

AD: Alzheimer's disease; Aβ: Amyloid-β; APP: Amyloid precursor protein; APP-CTFs: Amyloid precursor protein C-terminal fragments; BACE: β-secretase; F-Aβ: Fibrillar-Aβ42; FAD: Familial AD; 5xFAD: Mice containing 5 FAD mutations; LTP: Long-term potentiation; MOAB-2: Monoclonal antibody-2; O-Aβ42: Oligomeric Aβ42, PS: Presenilin; SDS-PAGE: Sodium dodecyl sulphate polyacrylamide gel electrophoresis; Tg: Transgenic; U-Aβ: Unaggregated-Aβ; WT: Wild type; IHC: Immunohistochemistry.

## Competing interests

The authors declare that they have no competing interests.

## Authors' contributions

KLY: Generated Figures 2B, 3A, B and 3C, and Figures 5A and 5B in collaboration with LMT. Contributed to interpretation of the results and preparation of the manuscript. LMT: Generated supplementary Figure 1A, Figures 3A, B and 3C, and 5A and 5B in collaboration with KLY. Contributed to interpretation of the results and prepared the manuscript in collaboration with MJLD. TK: Generated Figure 4A and 4B, and contributed to interpretation of the results. SCM: DAB analyses in Figure 2C. WBS: Performed the initial identification and characterization of MOAB-2, including Figure 1A, C and 1D, in collaboration with AMM. EN-H: Generated Figure 2A, supplementary Figure 1B, and contributed to revision of the manuscript. AMM: Contributed to initial identification and characterization of MOAB-2, including Figures 1A, C and 1D, in collaboration with WBS. YF: Initially generated and continues production of MOAB-2. SR: Provided HEK-APP_Swe_/BACE1 and detailed protocol for Figure 2A. WE: Provided 5xFAD tissue for IHC analysis and contributed to interpretation of the results. LB: Participated in the design of the study, particularly the generation of MOAB-2 antibody, contributed to interpretation of the results and manuscript preparation. GB: Participated in study design, contributed to interpretation of the results and preparation of the manuscript and revision. CY: Carried out immunoprecipitations in Figure 1E. Participated in study design, contributed to interpretation of results, and manuscript preparation. DMH: Provided substantial contributions to project conception, experimental design and manuscript preparation and revision. MJLD: Provided fundamental contributions to project conception, experimental design, interpretation of results, writing and revision of the manuscript. All authors read and approved the final manuscript.

## Supplementary Material

Additional file 1 **Figure S1**. MOAB-2 epitope mapping. **(A) **Peptide array (PepSpots™) consisting of a series of overlapping 10-mers from the -4 position of the Aβ sequence to residue 46. Membranes were incubated with MOAB-2 or IgG_2b _control antibody (1:2000 dilution from 0.2 mg/ml stock). Image representative of n = 3. **(B) **Dot blot of serial dilutions of rat (R) Aβ, human (H) Aβ40 and human Aβ42 probed with MOAB-2. Antibody concentration for MOAB-2 = 100 ng/ml (1:5000 dilution). Click here for file

## References

[B1] Lue LF Kuo YM Roher AE Brachova L Shen Y Sue L Beach T Kurth JH Rydel RE Rogers J Soluble amyloid beta peptide concentration as a predictor of synaptic change in Alzheimer's disease Am J Pathol 1999 155 853 862 10.1016/S0002-9440(10)65184-X 10487842 PMC1866907

[B2] Tomic JL Pensalfini A Head E Glabe CG Soluble fibrillar oligomer levels are elevated in Alzheimer's disease brain and correlate with cognitive dysfunction Neurobiol Dis 2009 35 352 358 10.1016/j.nbd.2009.05.024 19523517 PMC2725199

[B3] Gouras GK Tsai J Naslund J Vincent B Edgar M Checler F Greenfield JP Haroutunian V Buxbaum JD Xu H Intraneuronal Abeta42 accumulation in human brain Am J Pathol 2000 156 15 20 10.1016/S0002-9440(10)64700-1 10623648 PMC1868613

[B4] Thal DR Capetillo-Zarate E Del Tredici K Braak H The development of amyloid beta protein deposits in the aged brain Sci Aging Knowledge Environ 2006 2006 re1 10.1126/sageke.2006.6.re1 16525193

[B5] Christensen DZ Schneider-Axmann T Lucassen PJ Bayer TA Wirths O Accumulation of intraneuronal Abeta correlates with ApoE4 genotype Acta Neuropathol 2010 119 555 566 10.1007/s00401-010-0666-1 20217101 PMC2849938

[B6] Gouras GK Almeida CG Takahashi RH Intraneuronal Abeta accumulation and origin of plaques in Alzheimer's disease Neurobiol Aging 2005 26 1235 1244 10.1016/j.neurobiolaging.2005.05.022 16023263

[B7] Fernandez-Vizarra P Fernandez AP Castro-Blanco S Serrano J Bentura ML Martinez-Murillo R Martinez A Rodrigo J Intra- and extracellular Abeta and PHF in clinically evaluated cases of Alzheimer's disease Histol Histopathol 2004 19 823 844 15168346 10.14670/HH-19.823

[B8] D'Andrea MR Nagele RG Wang HY Lee DH Consistent immunohistochemical detection of intracellular beta-amyloid42 in pyramidal neurons of Alzheimer's disease entorhinal cortex Neurosci Lett 2002 333 163 166 10.1016/S0304-3940(02)00875-3 12429373

[B9] D'Andrea MR Nagele RG Wang HY Peterson PA Lee DH Evidence that neurones accumulating amyloid can undergo lysis to form amyloid plaques in Alzheimer's disease Histopathology 2001 38 120 134 10.1046/j.1365-2559.2001.01082.x 11207825

[B10] Busciglio J Pelsman A Wong C Pigino G Yuan M Mori H Yankner BA Altered metabolism of the amyloid beta precursor protein is associated with mitochondrial dysfunction in Down's syndrome Neuron 2002 33 677 688 10.1016/S0896-6273(02)00604-9 11879646

[B11] Gyure KA Durham R Stewart WF Smialek JE Troncoso JC Intraneuronal abeta-amyloid precedes development of amyloid plaques in Down syndrome Arch Pathol Lab Med 2001 125 489 492 11260621 10.5858/2001-125-0489-IAAPDO

[B12] Grundke-Iqbal I Iqbal K George L Tung YC Kim KS Wisniewski HM Amyloid protein and neurofibrillary tangles coexist in the same neuron in Alzheimer disease Proc Natl Acad Sci USA 1989 86 2853 2857 10.1073/pnas.86.8.2853 2649895 PMC287017

[B13] Masters CL Multhaup G Simms G Pottgiesser J Martins RN Beyreuther K Neuronal origin of a cerebral amyloid: neurofibrillary tangles of Alzheimer's disease contain the same protein as the amyloid of plaque cores and blood vessels EMBO J 1985 4 2757 2763 4065091 10.1002/j.1460-2075.1985.tb04000.x PMC554575

[B14] Billings LM Oddo S Green KN McGaugh JL Laferla FM Intraneuronal Abeta causes the onset of early Alzheimer's disease-related cognitive deficits in transgenic mice Neuron 2005 45 675 688 10.1016/j.neuron.2005.01.040 15748844

[B15] Casas C Sergeant N Itier JM Blanchard V Wirths O van der Kolk N Vingtdeux V van de Steeg E Ret G Canton T Massive CA1/2 neuronal loss with intraneuronal and N-terminal truncated Abeta42 accumulation in a novel Alzheimer transgenic model Am J Pathol 2004 165 1289 1300 10.1016/S0002-9440(10)63388-3 15466394 PMC1618627

[B16] Gouras GK Tampellini D Takahashi RH Capetillo-Zarate E Intraneuronal beta-amyloid accumulation and synapse pathology in Alzheimer's disease Acta Neuropathol 2010 119 523 541 10.1007/s00401-010-0679-9 20354705 PMC3183823

[B17] Lord A Kalimo H Eckman C Zhang XQ Lannfelt L Nilsson LN The Arctic Alzheimer mutation facilitates early intraneuronal Abeta aggregation and senile plaque formation in transgenic mice Neurobiol Aging 2006 27 67 77 10.1016/j.neurobiolaging.2004.12.007 16298242

[B18] Oakley H Cole SL Logan S Maus E Shao P Craft J Guillozet-Bongaarts A Ohno M Disterhoft J Van Eldik L Intraneuronal beta-amyloid aggregates, neurodegeneration, and neuron loss in transgenic mice with five familial Alzheimer's disease mutations: potential factors in amyloid plaque formation J Neurosci 2006 26 10129 10140 10.1523/JNEUROSCI.1202-06.2006 17021169 PMC6674618

[B19] Oddo S Caccamo A Shepherd JD Murphy MP Golde TE Kayed R Metherate R Mattson MP Akbari Y LaFerla FM Triple-transgenic model of Alzheimer's disease with plaques and tangles: intracellular Abeta and synaptic dysfunction Neuron 2003 39 409 421 10.1016/S0896-6273(03)00434-3 12895417

[B20] Oddo S Caccamo A Smith IF Green KN LaFerla FM A dynamic relationship between intracellular and extracellular pools of Abeta Am J Pathol 2006 168 184 194 10.2353/ajpath.2006.050593 16400022 PMC1592652

[B21] Takahashi RH Milner TA Li F Nam EE Edgar MA Yamaguchi H Beal MF Xu H Greengard P Gouras GK Intraneuronal Alzheimer abeta42 accumulates in multivesicular bodies and is associated with synaptic pathology Am J Pathol 2002 161 1869 1879 10.1016/S0002-9440(10)64463-X 12414533 PMC1850783

[B22] Wirths O Multhaup G Czech C Blanchard V Moussaoui S Tremp G Pradier L Beyreuther K Bayer TA Intraneuronal Abeta accumulation precedes plaque formation in beta-amyloid precursor protein and presenilin-1 double-transgenic mice Neurosci Lett 2001 306 116 120 10.1016/S0304-3940(01)01876-6 11403971

[B23] Wirths O Multhaup G Czech C Feldmann N Blanchard V Tremp G Beyreuther K Pradier L Bayer TA Intraneuronal APP/A beta trafficking and plaque formation in beta-amyloid precursor protein and presenilin-1 transgenic mice Brain Pathol 2002 12 275 286 12146796 10.1111/j.1750-3639.2002.tb00442.x PMC8095864

[B24] Christensen DZ Kraus SL Flohr A Cotel MC Wirths O Bayer TA Transient intraneuronal A beta rather than extracellular plaque pathology correlates with neuron loss in the frontal cortex of APP/PS1KI mice Acta Neuropathol 2008 116 647 655 10.1007/s00401-008-0451-6 18974993

[B25] Winton MJ Lee EB Sun E Wong MM Leight S Zhang B Trojanowski JQ Lee VM Intraneuronal APP, Not Free A{beta} Peptides in 3xTg-AD Mice: Implications for Tau versus A{beta}-Mediated Alzheimer Neurodegeneration J Neurosci 2011 31 7691 7699 10.1523/JNEUROSCI.6637-10.2011 21613482 PMC3118598

[B26] Oddo S Billings L Kesslak JP Cribbs DH LaFerla FM Abeta immunotherapy leads to clearance of early, but not late, hyperphosphorylated tau aggregates via the proteasome Neuron 2004 43 321 332 10.1016/j.neuron.2004.07.003 15294141

[B27] Christensen DZ Bayer TA Wirths O Formic acid is essential for immunohistochemical detection of aggregated intraneuronal Abeta peptides in mouse models of Alzheimer's disease Brain Res 2009 1301 116 125 19751708 10.1016/j.brainres.2009.09.014

[B28] LaFerla FM Green KN Oddo S Intracellular amyloid-beta in Alzheimer's disease Nat Rev 2007 8 499 509 10.1038/nrn2168 17551515

[B29] Horikoshi Y Sakaguchi G Becker AG Gray AJ Duff K Aisen PS Yamaguchi H Maeda M Kinoshita N Matsuoka Y Development of Abeta terminal end-specific antibodies and sensitive ELISA for Abeta variant Biochem Biophys Res Commun 2004 319 733 737 10.1016/j.bbrc.2004.05.051 15184044

[B30] Takeda K Araki W Akiyama H Tabira T Amino-truncated amyloid beta-peptide (Abeta5-40/42) produced from caspase-cleaved amyloid precursor protein is deposited in Alzheimer's disease brain FASEB J 2004 18 1755 1757 15364896 10.1096/fj.03-1070fje

[B31] Philipson O Lannfelt L Nilsson LN Genetic and pharmacological evidence of intraneuronal Abeta accumulation in APP transgenic mice FEBS Lett 2009 583 3021 3026 10.1016/j.febslet.2009.08.009 19683527

[B32] Stine WB Jr Dahlgren KN Krafft GK LaDu MJ In vitro characterization of conditions for amyloid-beta peptide oligomerization and fibrillogenesis J Biol Chem 2003 278 11612 11622 10.1074/jbc.M210207200 12499373

[B33] Dahlgren KN Manelli AM Stine WB Jr Baker LK Krafft GA LaDu MJ Oligomeric and fibrillar species of amyloid-beta peptides differentially affect neuronal viability J Biol Chem 2002 277 32046 32053 10.1074/jbc.M201750200 12058030

[B34] Fardilha M Vieira SI Barros A Sousa M Da Cruz e Silva OA Da Cruz e Silva EF Differential distribution of Alzheimer's amyloid precursor protein family variants in human sperm Ann N Y Acad Sci 2007 1096 196 206 10.1196/annals.1397.086 17405931

[B35] Henriques AG Vieira SI Crespo-Lopez ME Guiomar de Oliveira MA da Cruz e Silva EF da Cruz e Silva OA Intracellular sAPP retention in response to Abeta is mapped to cytoskeleton-associated structures J Neurosci Res 2009 87 1449 1461 10.1002/jnr.21959 19105196

[B36] Lee EB Leng LZ Zhang B Kwong L Trojanowski JQ Abel T Lee VM Targeting amyloid-beta peptide (Abeta) oligomers by passive immunization with a conformation-selective monoclonal antibody improves learning and memory in Abeta precursor protein (APP) transgenic mice J Biol Chem 2006 281 4292 4299 10.1074/jbc.M511018200 16361260

[B37] Vassar R Bennett BD Babu-Khan S Kahn S Mendiaz EA Denis P Teplow DB Ross S Amarante P Loeloff R Beta-secretase cleavage of Alzheimer's amyloid precursor protein by the transmembrane aspartic protease BACE Science 1999 286 735 741 10.1126/science.286.5440.735 10531052

[B38] Youmans KL Leung S Zhang J Maus E Baysac K Bu G Vassar R Yu C LaDu MJ Amyloid-beta42 alters apolipoprotein E solubility in brains of mice with five familial AD mutations J Neurosci Methods 2011 196 51 59 10.1016/j.jneumeth.2010.12.025 21219931 PMC3049315

[B39] Bayer TA Wirths O Intracellular accumulation of amyloid-Beta - a predictor for synaptic dysfunction and neuron loss in Alzheimer's disease Front Aging Neurosci 2010 2 8 20552046 10.3389/fnagi.2010.00008 PMC2879032

[B40] Hardy JA Higgins GA Alzheimer's disease: the amyloid cascade hypothesis Science 1992 256 184 185 10.1126/science.1566067 1566067

[B41] Haass C Selkoe DJ Soluble protein oligomers in neurodegeneration: lessons from the Alzheimer's amyloid beta-peptide Nat Rev Mol Cell Biol 2007 8 101 112 10.1038/nrm2101 17245412

[B42] Hardy J Selkoe DJ The amyloid hypothesis of Alzheimer's disease: progress and problems on the road to therapeutics Science 2002 297 353 356 10.1126/science.1072994 12130773

[B43] Giuffrida ML Caraci F Pignataro B Cataldo S De Bona P Bruno V Molinaro G Pappalardo G Messina A Palmigiano A Beta-amyloid monomers are neuroprotective J Neurosci 2009 29 10582 10587 10.1523/JNEUROSCI.1736-09.2009 19710311 PMC6665714

[B44] Walsh DM Selkoe DJ A beta oligomers - a decade of discovery J Neurochem 2007 101 1172 1184 10.1111/j.1471-4159.2006.04426.x 17286590

[B45] Wilcox KC Lacor PN Pitt J Klein WL Abeta oligomer-induced synapse degeneration in Alzheimer's disease Cell Mol Neurobiol 2011 31 939 948 10.1007/s10571-011-9691-4 21538118 PMC3146579

[B46] Klaver AC Patrias LM Finke JM Loeffler DA Specificity and sensitivity of the Abeta oligomer ELISA J Neurosci Methods 2011 195 249 254 10.1016/j.jneumeth.2010.12.001 21163305

[B47] Amadoro G Corsetti V Ciotti MT Florenzano F Capsoni S Amato G Calissano P Endogenous Abeta causes cell death via early tau hyperphosphorylation Neurobiol Aging 2011 32 969 990 10.1016/j.neurobiolaging.2009.06.005 19628305

[B48] Braak H Thal DR Ghebremedhin E Del Tredici K Stages of the pathologic process in Alzheimer disease: age categories from 1 to 100 years J Neuropathol Exp Neurol 2011 70 960 969 10.1097/NEN.0b013e318232a379 22002422

[B49] McLean D Cooke MJ Wang Y Fraser P George-Hyslop PS Shoichet MS Targeting the amyloid-beta antibody in the brain tissue of a mouse model of Alzheimer's disease J Control Release 2011 in press PMID:22245684 10.1016/j.jconrel.2011.12.036 22245684

[B50] Gouras GK Tampellini D Takahashi RH Capetillo-Zarate E Intraneuronal beta-amyloid accumulation and synapse pathology in Alzheimer's disease Acta Neuropathol 2010 119 523 541 10.1007/s00401-010-0679-9 20354705 PMC3183823

[B51] Mochizuki A Tamaoka A Shimohata A Komatsuzaki Y Shoji S Abeta42-positive non-pyramidal neurons around amyloid plaques in Alzheimer's disease Lancet 2000 355 42 43 10.1016/S0140-6736(99)04937-5 10615894

[B52] Aoki M Volkmann I Tjernberg LO Winblad B Bogdanovic N Amyloid beta-peptide levels in laser capture microdissected cornu ammonis 1 pyramidal neurons of Alzheimer's brain Neuroreport 2008 19 1085 1089 10.1097/WNR.0b013e328302c858 18596605

[B53] Walsh DM Tseng BP Rydel RE Podlisny MB Selkoe DJ The oligomerization of amyloid beta-protein begins intracellularly in cells derived from human brain Biochemistry 2000 39 10831 10839 10.1021/bi001048s 10978169

[B54] Abramowski D Rabe S Upadhaya AR Reichwald J Danner S Staab D Capetillo-Zarate E Yamaguchi H Saido TC Wiederhold KH Transgenic Expression of Intraneuronal Abeta42 But Not Abeta40 Leads to Cellular Abeta Lesions, Degeneration, and Functional Impairment without Typical Alzheimer's Disease Pathology J Neurosci 2012 32 1273 1283 10.1523/JNEUROSCI.4586-11.2012 22279212 PMC6796269

[B55] Wegiel J Kuchna I Nowicki K Frackowiak J Mazur-Kolecka B Imaki H Mehta PD Silverman WP Reisberg B Deleon M Intraneuronal Abeta immunoreactivity is not a predictor of brain amyloidosis-beta or neurofibrillary degeneration Acta Neuropathol 2007 113 389 402 10.1007/s00401-006-0191-4 17237937 PMC1824787

[B56] Golde TE Janus C Homing in on intracellular Abeta? Neuron 2005 45 639 642 10.1016/j.neuron.2005.02.013 15748837

[B57] Bittner T Fuhrmann M Burgold S Ochs SM Hoffmann N Mitteregger G Kretzschmar H LaFerla FM Herms J Multiple events lead to dendritic spine loss in triple transgenic Alzheimer's disease mice PLoS One 2010 5 e15477 10.1371/journal.pone.0015477 21103384 PMC2982845

[B58] Binder LI Frankfurter A Rebhun LI The distribution of tau in the mammalian central nervous system J Cell Biol 1985 101 1371 1378 10.1083/jcb.101.4.1371 3930508 PMC2113928

[B59] Ghoshal N Garcia-Sierra F Fu Y Beckett LA Mufson EJ Kuret J Berry RW Binder LI Tau-66: evidence for a novel tau conformation in Alzheimer's disease J Neurochem 2001 77 1372 1385 10.1046/j.1471-4159.2001.00346.x 11389188

[B60] Brown KD Binder LI Identification of the intermediate filament-associated protein gyronemin as filamin. Implications for a novel mechanism of cytoskeletal interaction J Cell Sci 1992 102 19 30 1500439 10.1242/jcs.102.1.19

[B61] O'Nuallain B Wetzel R Conformational Abs recognizing a generic amyloid fibril epitope Proc Natl Acad Sci USA 2002 99 1485 1490 10.1073/pnas.022662599 11818542 PMC122217

