# Supplementary figures and images for: Intraneuronal Aβ detection in 5xFAD mice by a new Aβ-specific antibody
Source: Mol Neurodegener. 2012 Mar 16;7:8. doi: 10.1186/1750-1326-7-8 (PMC3355009; doi:10.1186/1750-1326-7-8)

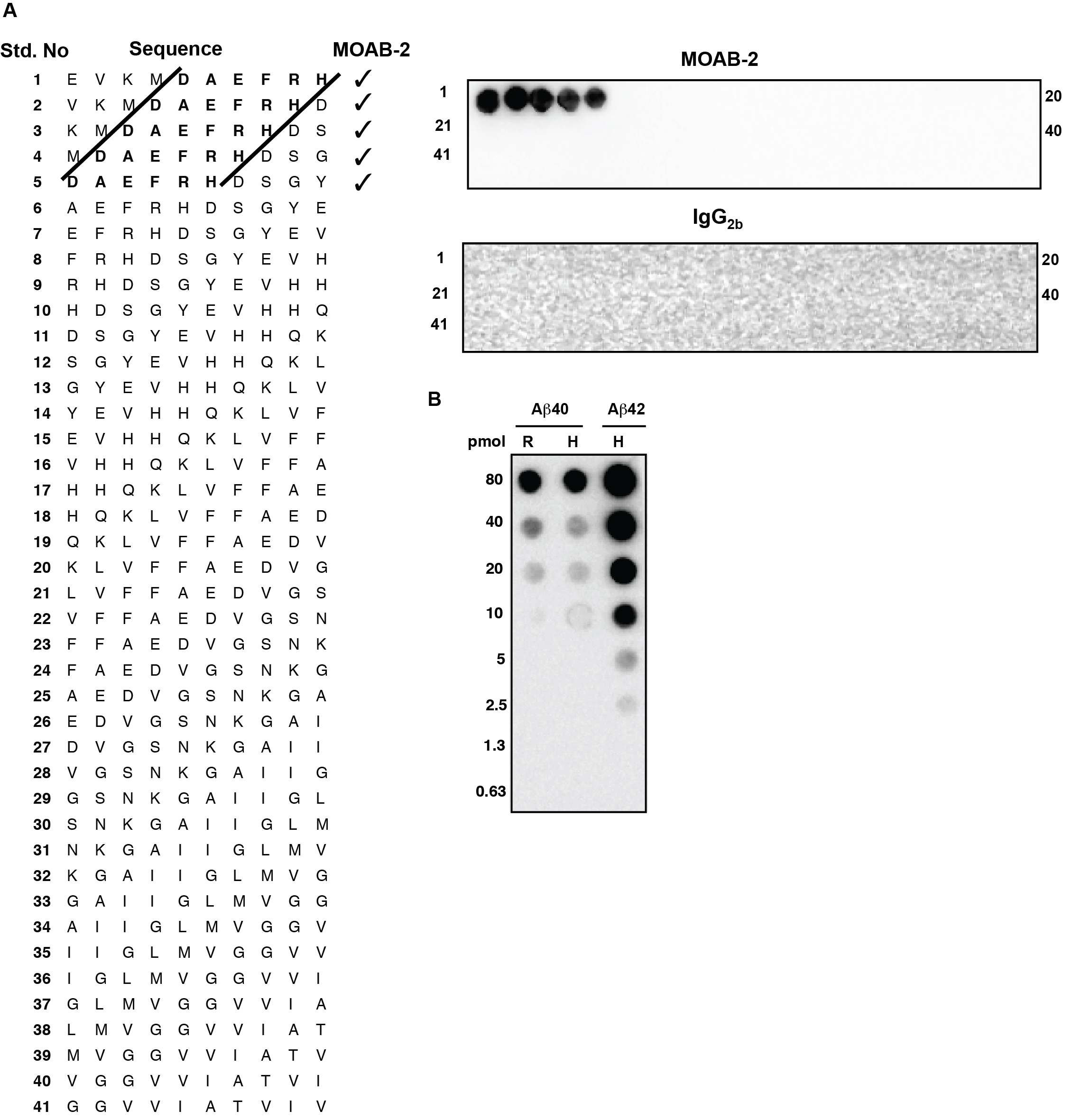

Supplement: Additional file 1 — Figure S1. MOAB-2 epitope mapping. (A) Peptide array (PepSpots™) consisting of a series of overlapping 10-mers from the -4 position of the Aβ sequence to residue 46. Membranes were incubated with MOAB-2 or IgG2b control antibody (1:2000 dilution from 0.2 mg/ml stock). Image representative of n = 3. (B) Dot blot of serial dilutions of rat (R) Aβ, human (H) Aβ40 and human Aβ42 probed with MOAB-2. Antibody concentration for MOAB-2 = 100 ng/ml (1:5000 dilution). [file 1750-1326-7-8-S1.TIFF]
